# Zero Tolerance for Coercion? Historical, Cultural and Organisational Contexts for Effective Implementation of Coercion-Free Mental Health Services around the World

**DOI:** 10.3390/healthcare11212834

**Published:** 2023-10-27

**Authors:** Richard Whittington, Deborah Oyine Aluh, Jose-Miguel Caldas-de-Almeida

**Affiliations:** 1 Centre for Research & Education in Security, Prisons and Forensic Psychiatry, Forensic Department Østmarka, St. Olav’s Hospital, 7030 Trondheim, Norway; 2 Department of Mental Health, Norwegian University of Science & Technology (NTNU), 7034 Trondheim, Norway; 3 Department of Public Health, Policy & Systems, University of Liverpool, Liverpool L69 3BX, UK; 4 Lisbon Institute of Global Mental Health, Comprehensive Health Research Centre, Nova Medical School, 1169-056 Lisbon, Portugal; 5 Department of Clinical Pharmacy and Pharmacy Management, University of Nigeria Nsukka, Nsukka 410105, Nigeria

**Keywords:** coercion, restraint, mental health services, implementation science

## Abstract

Coercion of service users/patients when receiving care and treatment has been a serious dilemma for mental health services since at least the 18th century, and the debate about how best to minimise or even eradicate compulsion remains intense. Coercion is now, once again and rightly, at the top of the international policy agenda and the COST Action ‘FOSTREN’ is one example of a renewed commitment by service user advocates, practitioners and researchers to move forward in seriously addressing this problem. The focus of service improvement efforts has moved from pure innovation to practical implementation of effective interventions based on an understanding of the historical, cultural and political realities in which mental health services operate. These realities and their impact on the potential for change vary between countries across Europe and beyond. This article provides a novel overview by focusing on the historical, cultural and political contexts which relate to successful implementation primarily in Europe, North America and Australasia so that policy and practice in these and other regions can be adopted with an awareness of these potentially relevant factors. It also outlines some key aspects of current knowledge about the leading coercion-reduction interventions which might be considered when redesigning mental health services.

## 1. Introduction

The Council of Europe [1] recently adopted a resolution which called for national governments “to immediately start to transition to the *abolition* of coercive methods in mental health settings” (italics added). This bold statement is the strongest ever inter-governmental pronouncement on this topic and indicates that, for international policy-makers at least, acceptance of coercion in mental health services is at an end. What used to be zero tolerance for violence by the recipients of healthcare [2,3] has swung around completely and become zero tolerance for unacceptable practices by the providers of healthcare when dealing with such violence, actual or perceived. However, how practical is it to aim to create mental health services that are totally free of coercion? Additionally, if it is achievable, how can hard-pressed services operating in real-world environments be helped to actually obtain to this goal? The COST Action ‘FOSTREN’ (www.fostren.eu, accessed on 18 October 2023) is a network of service user advocates, practitioners and researchers which is attempting to address these questions. In this paper we will examine the historical and contemporary social context in which decisions to use coercion occur to understand some of the barriers to change which have been identified following discussions within this network and more widely. We will also attempt to provide a state-of-the-art summary of current evidence for interventions which are potentially effective in at least minimising coercion, if not abolishing it. These interventions will be considered from an implementation science perspective because, beyond innovation, embedding sustained best practice in everyday care is likely to be the next challenge in transforming services.

## 2. Historical Context

Throughout history, attitudes toward the use of coercive measures in the treatment of persons with mental disorders were always closely associated with the prevailing cultural, religious and political context. If we go back in time, we can find, as far back as the 13th and 14th centuries, examples of places in Europe where people with mental disorders were treated humanely and integrated into the community. In Geel, Belgium, persons with mental disorders began, in those days, to be accepted in the homes of farmers, where they lived as members of the family and participated in work and community activities, a practice that has survived until today [4]. In Granada, Spain, in the 14th century, at the Maristan hospital, the first hospital created for the mentally ill in Europe, the patients were treated in a setting of tolerance and inclusion, which reflected the context that prevailed in that city at the time, marked by a peaceful and harmonious coexistence between Arabs, Christians and Jews that ushered a period of great progress in arts and science [5].

However, these examples were exceptions. In most places in Europe, for centuries, persons with mental disorders were isolated, chained and victims of all kinds of violence and abuse [6]. It was only in Greece that madness was viewed, for the first time, as a pathologic condition determined by natural causes. Different disorders were described, natural explanations were advanced for these disorders, and various therapeutic interventions were developed to mitigate the effects of the disorders. However, in Greece as well as in the Roman Empire, this medical interpretation always co-existed with magic and religious interpretations from the past. Those primarily responsible for caring for these persons were their families, who often limited themselves to isolating them and locking them up in their homes. The use of chains and other coercive instruments was common practice [7]. In the Middle Ages, care for persons with mental disorders continued to depend mainly on families, but in the most serious cases, when they were not abandoned and doomed to wander along with other beggars, they were locked up in cells located in hospices managed by monasteries and churches or in prisons administered by civil authorities [7]. The growing influence of the Christian faith in this period also explains the occurrence of regular pilgrimages, involving thousands of mentally ill every year, to shrines believed to have miraculous powers in healing people with mental illnesses.

At the beginning of industrialization, only patients with a lot of resources were treated in small private institutions, known in England then as “madhouses”. The poor did not have access to specialized institutions. The vast majority were locked up in their homes, placed in rooms of general hospitals or asylums, together with physical patients, elderly persons, and persons with disabilities, in subhuman living conditions. Others wandered aimlessly or ended up in workhouses.

The increase in the influx of patients of various types to these institutions and the growing denunciations of the terrible conditions in which they lived led to the creation, from the 18th century on, of new general hospitals in many cities in Europe and the appearance of institutions specially dedicated to various types of illnesses [8]. The asylums for the “insane” (as persons with mental disorders were designated then) that began to emerge in the 18th century and the first attempts to change attitudes towards mental illness were part of this movement [7]. These changes, however, would not have been possible without the new ideas of the philosophers of the Enlightenment. It was these new ideas that contributed to lessening the grip of superstition and emphasising a rational approach to natural phenomena, thus making possible the dissemination of new ways of understanding mental disorders and their treatment.

François Pinel, freeing the patients from the chains in Bicêtre in 1793, went down in history as the symbol of a new attitude towards the mentally ill and the author of the so-called “moral treatment”. However, similar innovations were at the same time being developed in other countries, and the concept of moral treatment was, in fact, first formulated by William Tuke in the United Kingdom, where he created The Retreat in 1792, an institution where the mentally ill were treated using moral management and with a minimum of mechanical restraint [9]. At the Lincoln Hospital, Robert Gardiner Hill also managed patients without any use of mechanical restraint. John Connolly, at the Hanwell Hospital, not only abolished the use of restraint but also introduced many other ideas, then quite innovative, such as improving facilities and food and promoting occupational activities; and, in Italy, Chiarugi also promoted the abolition of chains at the Ospedale Bonifazio in 1788 [6].

Anyway, Pinel had the merit of having defended in a very clear way that “the insane, far from being persons guilty of something which should be punished, are patients whose painful condition deserves all the consideration that is due to the humanity that suffers and for whom one must seek by the simplest means to restore the “deviated reason” [10]. The “moral treatment” proposed by Pinel was based on the conviction that it would be possible to introduce significant changes in the behaviour of patients via humane, though firm, attitudes on the part of the caregivers. Pinel’s innovation, however, did not consist only in this therapeutic approach to patients but in the association of this humanist stance with, on the one hand, the effort to study the phenomenon of madness rationally and methodically and, on the other hand, the recognition of the importance of the patient’s subjectivity [11].

The new therapeutic approaches based on moral treatment played a fundamental role in the development of the psychiatric asylum model that would form the basis of psychiatric care throughout the world since the beginning of the 19th century. At the same time, the recognition of the need to study mental illnesses from a scientific perspective would allow the birth of psychiatry as a medical discipline and contribute to the developments that would come to be verified in the understanding of the causes of mental illnesses at a biological, psychological and social level.

Although this new approach had a significant impact in many countries, the release of shackles and the prohibition of systematic punishment did not imply the abandonment of compulsion and other forms of coercion. All people hospitalized in asylums continued to be committed involuntarily and had to be certified as mentally ill, which explains why most European countries began to create legislation to regulate psychiatric hospitalization during the 19th century. Moreover, voluntary hospitalizations only started around 1930, and the large psychiatric institutions, separated from the general health system, remained the mainstay of mental health care for much longer.

The truth is that it took more than a century and two world wars for the understanding to develop those asylums also had important limitations in effectively responding to the needs of people with mental disorders and to realize that to end the marginalization and exclusion of persons with mental disorders, it was necessary to develop new models of care, better respond to the various needs of the mentally ill, and promote their access to equal rights.

World War II and the impact of the Holocaust [12,13], responsible for the killing of hundreds of thousands of mentally ill persons in psychiatric hospitals, were the great turning point in this evolution. It was this impact born out of the horrors of the war and the experiences of solidarity with its victims that created the conditions for the emergence of a new recognition of the importance of respecting the political, social and economic rights of all individuals, including the ones belonging to the most vulnerable and excluded groups. This impact was also fundamental for the emergence of two movements that would constitute, and continue to do so until today, an essential driver of the fight against the use of coercion: the reform of mental health services and the international human rights mechanisms.

### 2.1. Post-War Health Service Reforms

Both in Europe and in the US, the contestation against psychiatric asylums grew significantly after the war, contributing to the implementation of mental health service reforms based on three common elements: deinstitutionalization and development of services in the community, involvement of primary health care; and protection of the human rights of persons with mental disorders.

These reforms were inseparable from other social movements that sprung up at the same time with the objective of fighting for the emancipation of groups that had been discriminated against and excluded until then because of their gender, sexual orientation or ethnicity. As Franco Basaglia, the leader of the Italian psychiatric reform, said: “when we say no to the asylum, we say no to the world’s misery, and we join all the people in the world who fight for the emancipation of human beings” [14]. It was not by chance that the first mental health services reforms were initiated in countries that were engaged in political processes aiming at improving the social and economic conditions of their populations and ensuring better access to health care. Similar reforms later developed in other countries (e.g., in Southern Europe, Latin America and Eastern Europe) started when these countries were experiencing processes of democratisation, as could be seen in Spain [15], Brazil and Chile [16] and the Czech Republic [17]. More recently, mental health reforms were also developed in other parts of the world: among others, in Lebanon [18], India [19] and South Africa [20].

### 2.2. Key Statements on Human Rights since 1945

The Universal Declaration of Human Rights (UDHR), adopted by the United Nations General Assembly in 1948 in response to the atrocities committed during World War II, marked the beginning of a movement that led to the creation of a comprehensive set of international human rights mechanisms, which had, and still have, a key role in the attitudes toward the use of coercion in mental health services.

The UDHR does not mention mental health, but Article 25 says that: “Everyone has the right to a standard of living adequate for the health and well-being of themselves and of their family, including food, clothing, housing and medical care and necessary social services, and the right to security in the event of unemployment, sickness, disability, widowhood, old age or other lack of livelihood in circumstances beyond his control” [21].

The first official document that directly and explicitly refers to mental health is the WHO definition of health in 1948 as “a state of complete physical, mental and social well-being [which] is not just the absence of illness or infirmity” [22]. The International Covenant on Economic, Social and Cultural Rights [23] is especially important because it recognizes the right of all persons to enjoy the “best possible physical and mental health status”, and because, for the first time, the right to health is recognised in a legally binding pact.

Another document related to the rights of persons with mental disorders is the UN General Assembly Resolution “Principles for the protection of people with mental illness and the improvement of mental health care” [24], approved on 17 December 1991, which describes Fundamental Freedoms and Basic Rights—Access to quality care, Internationally recognized rights, Protection of dignity and prohibition of discrimination—as well as Principles related to consent, involuntary treatment regulation (decision, review, procedural safeguards), community life, and access to information, among others.

The most recent and most important international treaty that is relevant to the use of coercion is the Convention for the Rights of Persons with Disabilities (CRPD), approved in 2008 [25]. The purpose of the Convention “is to promote, protect and ensure the full and equal enjoyment of all human rights and fundamental freedoms for all persons with disabilities”, which includes persons with serious mental disorders.

The approval of the CRPD represents a great advance in the promotion of the rights of persons with mental disorders, as it is based on a general consensus of the international community (governments, NGOs and citizens) on the need to effectively ensure respect for the integrity, dignity and individual freedoms of persons with mental disabilities and to strengthen the prohibition of discrimination against these citizens via laws, policies and programs that specifically meet their needs and promote their participation in society. It includes several articles especially relevant to the reduction of coercion. Article 12, on equal recognition before the law, places the legal capacity of persons with disabilities on an equal footing with others. According to this article, people with disabilities have the right to maintain and exercise their legal right to make decisions and should have access to the support they may need in their decision-making process from people they trust who may be legally recognized or informally designated.

Per the CRPD Committee’s interpretation, any form of substitute decision-making is considered a violation of the Convention’s guarantee of legal capacity on an equal basis, which means that involuntary treatment is absolutely prohibited. This position is supported by other UN bodies and is not supported by others, a split that is also found among users and professionals [26,27] who, although believing that supported decision-making is, in general, the best way of respecting the rights of persons with mental conditions, consider that, with the mental health services currently available in most countries, it is impossible to completely rule out the use of substitute decision-making in every circumstance. However, despite these differences of interpretation on this specific point, there is now a broad consensus on the importance of joining efforts on concrete actions that may contribute to effectively reducing coercion in mental health services—at the policy and legislation level, the incorporation of evidence-based interventions in clinical practice, the reform of mental health systems, and research.

## 3. Contextual Factors Influencing Coercion

The prevalence of coercion in mental health care is influenced by various contextual factors that are interrelated and not necessarily associated directly with mental health professionals or service users. Although clinical and sociodemographic features of patients are well-established risk factors for the use of coercion [28,29], contextual factors such as service-related characteristics and characteristics of mental health care professionals appear to be influential as well [28,30,31,32,33,34,35]. Factors such as economic conditions and the impact of COVID-19 have the potential to augment the demand for mental health services. Additionally, the design, efficiency and staffing levels of these services play a crucial role in determining their ability to meet increased demands. Staff attitudes are significant determinants in the utilization of coercion, and these attitudes are typically aligned with prevailing mental health laws, policies, and public attitudes towards individuals with mental health conditions. It is crucial to recognize these contextual factors since they could guide the development and implementation of interventions to reduce coercion in specific settings. The contextual factors are particularly important to note since the strategies aimed at minimizing coercive practices in mental health settings are often developed and tested in controlled, idealized environments that do not fully mirror the complexities of many mental health systems. Consequently, the recommendations that arise from these studies are often too generic to effectively address the specific practical barriers that mental health professionals encounter when attempting to adapt these strategies to their particular organizational contexts. Moreover, they fail to account for changes that affect services, such as the effects of austerity measures and regulatory changes on public services [36]. Not many studies have specifically assessed the influence of contextual factors on the use of coercion [37].

### 3.1. Service Design

Considering that involuntary legal status at the time of admission predicts the use of other coercive measures [38,39,40], it seems reasonable to assume that contextual factors that influence involuntary admission indirectly contribute to the use of additional coercive measures such as restraints and seclusion. The organization of mental health services influences the quality of mental health care, and since the use of coercive measures is an indication of the quality of mental health treatment [41], the same variable indirectly influences it. The recommended approach for organizing mental health care services is to close large, independent psychiatric hospitals and instead establish psychiatric departments within general hospitals and community-based mental health services [42]. This model is preferred as it promotes continuity of care, increases user satisfaction, enhances human rights protection, and decreases the stigma surrounding mental health [42].

Deinstitutionalization is a sensitive process that needs to be managed with caution to avoid negative consequences on the quality of care, including the use of coercive measures. Decreasing psychiatric beds without a corresponding development of community resources could lead to adverse outcomes, such as a rise in involuntary admissions. An ecological study in England found a synchronous increase in the rate of involuntary admissions with decreasing available beds between 1988 and 2008 [43]. Smith et al. [44] also found that a decreased availability of psychiatric beds amplified the impact of austerity measures on the rates of involuntary admissions and Place of Safety detentions in England. In Italy, however, a reduction of psychiatric beds led to a reduction in the rates of involuntary admissions within the 18 years following the implementation of the new Mental Health Law [45]. The parallel expansion of community mental health care, the legal framework, and the nationwide movement for the rights of people with mental health conditions in Italy may explain the disparity in the impact of reducing psychiatric beds.

The availability of alternative services to inpatient care has been shown to significantly reduce the rates of involuntary admission in a given location [46], especially in intensive crisis teams in the community [47,48]. The extent to which the available mental health services are utilized has significant effects on the risk of involuntary admissions [49]. The importance of a functional primary care network that is adequately integrated into the mental health care system is highlighted by the association between referrals from general practitioners and a decreased incidence of involuntary admissions [42]. To meet the non-clinical needs of people with mental health conditions, social services that are complementary to the services offered by psychiatric care are required and have been reported to have a protective effect on acute psychiatry [50]. Further consideration of specific interventions associated with reduced coercion will be given in Section 3 below.

The use of restraints and seclusion is also known to be influenced by organizational factors, such as ward characteristics. Wards located in metropolitan areas have been reported to use more isolation and restraint [51], possibly due to higher demand for services. Ward size, male-to-female staff ratio, and staff experience are also linked to the use of seclusion [52,53,54]. In addition, factors like staffing levels, staff training, and attitudes are connected to the use of coercive measures. Nurses from countries with a varying range of resources report how insufficient resources, especially human resources, contribute to the use of coercive measures [55,56,57,58,59]. Staffing levels have been reported to be related to perceived concerns about the causes and responses to conflict and aggression [60]. However, it is possible that increasing staffing levels in a ward may not necessarily lead to a reduction in the use of coercion, as a recent study reported that both seclusion and mechanical restraint were utilized more frequently inwards with more nurses [61].

### 3.2. National Legislation and Policy

The use of coercive measures in mental health care can also be viewed as an indicator of the underlying characteristics of mental health legislation and policies in a specific jurisdiction. Studies on the impact of legislation on the rates of involuntary admission have not shown consistent results, and the most effective legal framework that can safeguard the rights of people with mental health conditions and the general public while also minimizing coercion remains uncertain. In Switzerland and Italy, new mental health legislations were associated with lower proportions of involuntary admissions [62], while the reverse was the case in China [63]. The increase in involuntary admission rates after a change in mental health legislation in China was attributed to the comparatively low number of psychiatric beds and the cultural stigma there. A review of the mental health laws in European countries revealed that those that demanded the participation of independent legal representatives had lower involuntary admission rates [64]. However, a more recent comparison of involuntary admission rates in 22 countries found no association with any characteristics of the legal framework [65]. Cultural norms have an impact on the laws regulating certain coercive measures, which may explain why some restrictive practices are legally regulated and monitored in some countries but not in other countries.

While involuntary admissions are regulated by mental health legislation in most European countries, the use of mechanical restraints, seclusion, and other coercive measures is not regulated by law in some countries and is left to the discretion of hospitals and regional authorities [55]. An international evaluation of restraints conducted more than ten years ago indicated that there was insufficient information available and that national databases were necessary [66]. Differences in the prevalence of restraints used in Dutch and British services were attributed to Dutch psychiatric professionals’ opinion of involuntary medication being more invasive and threatening to personal integrity to a higher degree than the application of seclusion or mechanical restraint. This is reflected by the Dutch mental health legislation, which is very restrictive regarding involuntary medication, permitting its use only in cases of acute emergency [66]. In traditional acute settings in the UK, physical restraints tend to be preferred over mechanical restraints, which are rarely used. However, if mechanical restraints are used, they are carefully observed and recorded [66,67]. This has important implications since mandatory review of all mechanical restraint episodes has been reported to be associated with low rates of use [68], and hospitals using detailed guidelines for seclusion and restraint have been reported to use fewer of these coercive measures [69].

### 3.3. Economic Factors

Economic recessions and austerity measures often lead to an increased need for mental health care, which can have major effects on individuals and society. A study conducted in Florida confirmed that declining regional economies reduce community tolerance for people seen as a threat to others, leading to an increase in involuntary psychiatric admissions [70]. Similarly, in the UK, the rise in involuntary admissions has been attributed to the economic recession, legislative changes, and the impact of austerity measures on health and social care services [44]. Previous studies have highlighted the two opposing trends of funding reductions with ensuing staff shortages and growing demand to minimize the use of coercive measures notwithstanding these insufficiencies [60,71]. These pressures have been linked to the emergence of “defensive psychiatry”, a term coined to describe the practice of healthcare professionals making decisions to reduce their legal liability while ensuring the safety of patients and others. This phenomenon has been reported among both psychiatrists and nurses [72,73].

### 3.4. COVID-19

The impact of the COVID-19 pandemic on the use of coercive measures has been contradictory across different countries, as some have documented changes during lockdowns while others have reported no changes. In Portugal, although there was a decrease in overall psychiatric admissions, the proportion of involuntary admissions increased during the pandemic [74]. In Italy, all psychiatric admission rates significantly decreased in the 40 days after the start of the pandemic, but involuntary admission rates remained unchanged [75]. In Canada, a reduction in the use of restraints and seclusion was reported during the COVID-19 pandemic due to organizational changes that were made to foster service users’ well-being [76], while in Germany, an increase in these coercive measures was reported during the pandemic due to rules made to prevent infection [77].

### 3.5. Staff Attitudes and Training

The duration and frequency of restrictive practices vary across countries, organizations, and individuals, and this cannot be explained solely by differences in legislation [78,79,80]. Evidence increasingly suggests that staff attitudes, particularly those of nurses, could have a direct impact on the prevalence and continued use of restrictive practices [51,81]. The variation in the thresholds for using restrictive practices among staff members [82] implies that there are differences in how staff perceive violence [83] and how much agitation and disturbed behaviour they are willing to tolerate [84]. It is challenging to determine the actual need for restrictive practices when they are used as routine procedures rather than as a last resort for safety purposes [85]. Nurses often have to make decisions about restrictive practices, which can result in inconsistency and an underestimation or exaggeration of risk, leading to unnecessary or prolonged seclusion or restraint [86]. These individual differences were highlighted as reasons to avoid using ward culture as an explanation for the adoption of restrictive measures in psychiatric wards [80]. Since each staff member has a unique perspective on aggressiveness, training for dealing with aggression or violent occurrences should be done, at least in part, on an individual basis. A lack of training on the alternative approaches to coercion is also implicated in the use of coercion, as many mental health professionals tend to perceive coercion as an inherent part of care [78]. This argument is supported by evidence highlighting the effectiveness of training in reducing the occurrence of coercion [87].

### 3.6. Public Attitudes

Public attitudes towards coercive measures in psychiatric care are important in understanding the context of their use. These attitudes can range from stigmatizing and fear-based perceptions of people with mental health conditions to more empathetic and supportive attitudes that prioritize human rights and recovery-oriented care. Cultural factors can also play a role, as collectivist cultures often emphasize family involvement in decision-making, while individualistic cultures may prioritize individual autonomy and self-determination [88]. Increasingly, there is a reduced tolerance for deviant behaviours, which can lead to pressure for inpatient care and the subsequent use of other coercive measures [89]. Empirical evidence suggests that public attitudes towards coercive measures vary by country and are influenced by existing laws and regulations. In Norway and France, the majority of participants agreed that involuntary hospitalization was acceptable in certain circumstances [90,91]. From 1993 to 2011 in Germany, the percentage of individuals who endorsed involuntary admissions remained steady despite the presence of mental health awareness initiatives and anti-stigma campaigns during that time. However, there was a decrease in opposition to involuntary admissions for reasons not aligned with legal criteria, suggesting a shift towards a more liberal perspective on patient rights while displaying reduced tolerance for inappropriate behaviour [92]. These findings highlight the necessity of incorporating specific elements pertaining to the rights of people with mental health conditions into stigma reduction campaigns. Similarly, a US study found that support for involuntary admissions increased steadily over the years for various mental health conditions [93].

Measuring the impact of these contextual factors is challenging; nonetheless, they play a significant role in the problem and present possibilities for long-term interventions. Since contextual factors do not act independently and often reinforce one another, effective strategies to reduce coercion must address these contextual factors using multiple approaches. Given the involvement of various stakeholders, it is crucial not to leave the responsibility for reducing coercion in mental health care solely to mental health professionals. The complexity of the problem calls for collaborative efforts involving various stakeholders, such as policy-makers, mental health professionals, service users, and the broader community.

## 4. Current Issues: Innovation and Implementation

The growth in knowledge on contextual factors in coercion has led to a step-change in ideas and initiatives to tackle the issue. We are still nowhere near possessing a road map toward the ultimate goal, but there has been a noticeable shift in how much attention and money society as a whole is willing to pay to address the problem. It is too soon to say that there is zero tolerance for coercion in services, but judging at least by the growing attention devoted to considering the problem, there is certainly significant discomfort about the situation amongst many decision-makers, which differs from widespread obliviousness in some earlier periods of time. People with lived experience of mental disorders who have suffered more than discomfort when subjected to coercion may be frustrated by the slow pace of change, but nonetheless, there are grounds for optimism now that did not exist twenty years ago.

Such optimism can be based on awareness of, for example, international conventions noted above like the United Nations Convention on the Rights of Persons with Disabilities (CRPD) [25] and the European Convention for the Prevention of Torture and Inhuman or Degrading Treatment or Punishment (CPT) [94]. We should also be encouraged by high-level pronouncements about international healthcare policy, such as the resolution noted above adopted by the Council of Europe in 2019 “to immediately start to transition to the *abolition* of coercive methods in mental health settings” [1]; (italics added). These conventions and resolutions have undoubtedly caused huge tensions between moderate ‘coercion-reductionists’ and radical ‘coercion-abolitionists’, but regardless of this important debate, these agreements set a standard to which services can aspire. They also provide the basis for frameworks which can be used to develop best practices [95,96,97] and are a stimulus for wider society in each country to think seriously about the problem, its solution and the consequences.

Grand conventions and policy pronouncements have, of course, been made many times in the past without making much difference in this or any other area of human activity. The shift we are witnessing now is different, though, because it is underpinned and made possible by the new culture of applied science and evidence-based policy, which characterises contemporary healthcare planning in many countries. Whilst this ‘new public management’ [98] approach has many downsides, its emphasis on maximising effectiveness and minimising costs has led to the channelling of significant government funding into the veiled but costly topic of tackling coercion. This funding has supported a number of well-designed research studies at scale when appropriate and enabled attempts to translate their findings into clinical guidelines. In this sense, the hidden topic of coercion now sits alongside the headline problems of heart disease and cancer in the library of national guidelines curated by the National Institute of Health and Care Excellence (NICE) in England and equivalent organisations around the world [99]. The funding is minuscule compared to these high-profile disorders, but the new visibility of coercion as a topic is undeniable. This is especially noteworthy as coercion is a patient safety issue in which the health service itself causes harm [100] rather than being a health issue caused primarily by external forces (society, the individual, etc.), which the health service then tries to address. As an iatrogenic problem, the health service has the primary liability and accountability for the injuries and trauma caused by coercion and inevitably therefore national decision-makers might be tempted to avoid the issue.

Instead, as noted, relatively significant resources have been devoted over the past twenty years to understanding why coercion happens and how it can be avoided. Baker et al. [101] estimate that the amount of research conducted on this topic doubled across the first two decades of the current century. This research is often then rapidly and rigorously translated into practical guidance for clinicians operating on the front line. The first relevant national guideline in the UK, for example, was published in 2005 and expanded significantly for the second edition [99]. Several equivalent national guidelines are available from around the world (e.g., Germany: [102]). These guidelines are usually tied to specific outcomes and processes against which organisations are audited in an effort to ensure that they are actually implemented in practice [103]. Whilst such auditing is clearly burdensome for practitioners who are already overstretched in delivering care and treatment, they give authority to the guidelines and enable transparency, which is often welcomed by both service users and clinicians.

The point here is that the ‘pipeline’ running from scientific study to guidelines to auditing is relatively expensive and highly novel as a system-wide approach for tackling a problem like coercion, which is usually low-visibility and often completely hidden. After two decades, this programmatic application of science and policy is beginning to yield a consensus on what can be done by any organisation, which is as serious as it should be about starting to address coercion. Whilst the research literature since 2000 alone is vast, this evidence-based consensus is forming around a number of well-tested interventions which can be adopted or adapted by health service leaders and managers to change practice across a whole organisation or within a specific team.

### 4.1. Promising Innovations

Three recent systematic reviews, in combination, provide a comprehensive overview of the state-of-the-art in this area, which can support decision-makers in this task. All three reviews adopted a transparent and systematic approach to gathering relevant empirical evidence and focused on interventions to reduce in-patient coercion and/or community-based coercion (i.e., involuntary admission) in mental health services. Each had distinctive aims and adopted a different approach to analysing their included studies, but taken together, their conclusions on certain key aspects are in harmony and provide a credible basis for the next stage of clinical development and research.

Firstly, Gooding et al. [104] conducted a UN-commissioned scoping review of empirical studies reporting in the English language on interventions which have been tested in either in-patient or community settings around the world since 1990. They adopted a scoping review methodology which was systematic in identifying relevant studies but deliberately broad in terms of methodologies and outcomes which were included. Such a scoping approach works well in a field like coercion reduction, where key concepts remain unclearly defined [105], and there is a lack of any unifying theory. Thus, their approach is comprehensive and transparent but essentially exploratory rather than confirmatory. In contrast, Barbui et al. [87] conducted a much more focused meta-analysis restricted to randomised controlled trials which had either in-patient coercion (i.e., restraint) or involuntary admission as an outcome. This umbrella review of 23 primary studies published since 1996 in multiple languages synthesised the findings from existing systematic reviews and provides the most precise estimate of effect sizes for various interventions currently available. Thirdly, Baker et al. [101] conducted a mapping review which analysed the core components of 150 interventions designed to reduce the use of restrictive practices in adult mental health in-patient settings only. Like Gooding et al., they took a broadly inclusive methodological approach toward evaluation studies using any research design and published in English from 1999 onwards. By looking across these studies using a particular theoretical approach (Behaviour Change Techniques, BCT), they sought to identify the key ‘specific, irreducible, active’ ingredients of a successful intervention regardless of its specific packaging or branding. This approach recognises that whilst there are numerous more-or-less well-defined interventions in the published literature which may appear distinctive, they are likely to share a relatively small number of discrete elements (e.g., using instruction or reinforcement) which could be isolated and used to devise a theoretically based ‘ideal’ intervention.

All three reviews revealed a strong concentration of research in high-income countries and a further clustering of research in certain countries in Europe, North America and, to a lesser extent, Latin America. This clustering of research in countries with relatively plentiful financial resources for mental health services and research inevitably skews the applicability of the findings away from automatic adoption in low- and medium-income countries (LMICs) where, paradoxically, the problem of coercion is likely to be even greater. Gowda et al. [106], for example, reported that 20% of patients in a mental health service in India were restrained, and although international comparisons are very difficult to make, this is more than double the comparable rate in European services [107]. Whilst there are efforts currently underway to construct a shared terminology in this area [108], conclusions drawn from the evidence base must, therefore, always be made cautiously with an awareness of this significant cultural and economic variation.

In combination, the three reviews identify a huge array of interventions which have been developed with some intention or potential to reduce coercion in either in-patient or community settings. In combination, Gooding et al. [104] and Baker et al. [101] specify over 60 distinct interventions. Many interventions in this array, it must be said, are similar approaches with different packaging, which supports the approach adopted by Baker et al. [101] to uncover the core components across the huge diversity of available in-patient programmes. Whilst there is some overlap between their intervention lists and significant overlap in terms of underlying components (e.g., the use of some form of training), this suggests a highly creative area with many promising innovations.

From this large array, healthcare policymakers and health service leaders tasked with choosing the most appropriate and effective intervention to remodel their service away from a reliance on coercion need to know which approaches are emerging as strong contenders in this crowded field. Some tentative conclusions on this can be derived from looking across the three reviews here, and some of the most promising interventions are listed below in Table 1. It should be noted that inclusion in this table indicates that an intervention has, at the very least, been widely implemented based on the location of relevant publications reporting on the intervention. Inclusion here does not, however, on its own indicate that the intervention has been found to be effective or that it has even been tested in a trial. Those interventions with this additional significance are highlighted separately in Table.

A number of these interventions are worth considering in more detail.

#### 4.1.1. Six Core Strategies for Reducing Seclusion and Restraint

This approach was developed in the USA from 2002 onwards under the auspices of the National Association of State Mental Health Program Directors (NASMHPD) [109]. The strategies themselves are: Leadership Towards Organizational Change; Using Data to Inform Practice; Workforce Development; Use of Seclusion and Restraint Reduction Tools: Peer Roles in Inpatient Settings: and Use of Rigorous Debriefing Techniques. The model is designed specifically to prevent the conflicts and violence that lead to coercive practices and is directed ultimately to greatly reduce or eliminate the use of seclusion and restraint in mental health services. A public health prevention approach which distinguishes primary, secondary and tertiary levels of prevention is also adopted as a core component of this model. There is an ongoing emphasis on the potential for patient recovery (as a defence against therapeutic despair or pessimism), patient resilience and an awareness of the importance of previous trauma in current experiences of mental health care. A revised and current approved training curriculum has been developed with extensive manualized training, including video and text training materials, a Planning and Implementation Tool, a Debriefing Tool and an Inventory of Seclusion and Restraint Reduction Interventions (ISRRI) tool that can be used to audit implementation activities.

This intervention has been implemented and tested more widely than any other intervention in in-patient settings, with studies reported from the USA, where it originated, Canada, across Europe (including Poland, Spain, Norway, Finland, Ireland, Canada, Germany, Denmark and the UK) and Australia. All of the six evaluation studies identified by Gooding et al. [104] reported a substantial reduction in seclusion and restraint after implementation of this approach in child/adolescent, adult in-patient or forensic services. Moving beyond in-patient services, the approach has been used to stimulate the co-creation of a community-based intervention for the prevention of involuntary admission, which is currently being trialled in Norway [110]. It was not explicitly specified as an intervention by Barbui et al. [87], so a precise effect size cannot be estimated.

#### 4.1.2. Safewards

Safewards [111] is an approach developed more recently in the UK with a wider focus on conflicts which commonly occur in in-patient services between service users and staff rather than seclusion and restraint specifically. At the same time, it has a narrower focus on interpersonal interaction between staff and patients rather than organisational systems across an entire institution. The original trialled version of the intervention [112] involved ten specific measures to be utilised by staff drawn from the overall model. These include “mutually agreed and publicised standards of behaviour by and for patients and staff…a requirement to say something good about each patient at nursing shift handover…(and) a crate of distraction and sensory modulation tools to use with agitated patients (stress toys, mp3 players with soothing music, light displays, textured blankets, etc.)” [112].

This initial cluster RCT of Safewards in the UK recorded a 15% reduction in conflict in the intervention wards, but the methodology has attracted some criticism [113]. The reviews considered here do not have much further evidence on the effectiveness (as opposed to implementation) of Safewards, which may be due to it only becoming available for evaluation relatively recently (more than a decade after Six Core Strategies was first published) but recent trials with some promising findings have been conducted in Australia and Germany [114,115].

#### 4.1.3. Restraint Education

Barbui et al. [87] conclude that ‘staff training’ does have an association with reduced restraint use in in-patient settings, which can be precisely estimated based on twelve RCTs. The combined risk ratio was 0.74, with 95% confidence intervals well below 1. This evidence is stronger than that for either of the two interventions above, but the usefulness of the finding for decision-makers in mental health services is limited for various reasons. Firstly, all of the studies were conducted in some form of nursing home, and several of these were specifically for people with dementia. The applicability of these results to mental health services provided for younger adults and children with functional mental disorders is very questionable. Secondly, there are no details in the review on the content, format or duration of the training used in the various studies, so there is no consistent ‘package’ which can be adopted without further examination of the individual studies. If the methodology adopted in these training studies could be used to inform high-quality RCTs of detailed and consistent training ‘packages’ such as Safewards, then the evidence base of the latter would be swiftly enhanced.

#### 4.1.4. Joint Crisis Plans and Shared Decision-Making

Turning from in-patient to community services, there is evidence of a significant reduction in involuntary admissions from at least one RCT in the Barbui et al. [87] review. Henderson et al. [116] tested plans agreed between UK outpatients with psychotic disorders or non-psychotic bipolar disorder and their care teams. These plans included early warning signs for relapse and preferred forms of treatment during a crisis. Whilst the crisis plan intervention in this individual trial was effective, shared decision-making overall in the meta-analysis (with crisis plans as one example) had only a weak association with reduced compulsory admission across the six trials which tested it. Nevertheless, shared decision-making defined more broadly as “a formalised process…by which clinicians and consumers engage in a collaborative decision-making process for healthcare decisions” [117], has been widely implemented and evaluated in high and middle-income countries across Europe, North America, South America and the Middle East.

### 4.2. From Innovation to Implementation

Most of the interventions considered above are genuinely innovative, but this is not because they involve the discovery of previously unknown aspects of human experience and behaviour. Rather, their novelty lies in their structure and composition as protocol-driven packages of existing knowledge derived from research or clinical wisdom which have been constructed and, in some cases, repeatedly tested in real-world settings with more or less success. It is their operationalisation, integration and systematisation of management and clinical practices, which previously were deployed unreflectively, which is innovative and, as discussed, many of them have at least some evidence of effectiveness in terms of reducing various types of coercive measures.

Unfortunately, innovation alone in healthcare is rarely enough to ensure a sustained change in practice over long periods of time. Most complex human systems have in-built inertia acting against new interventions, and this can only be overcome with significant efforts, including both extra resources for transformation and a genuine commitment amongst those required to deploy the new intervention. Therefore, innovation requires, in addition, an understanding of implementation, which raises a whole different set of questions from those answered in a successful effectiveness trial. It has been said [118] that, on average, it takes up to 20 years for an innovation to be adopted into everyday practice, and 50% of innovations fail to transfer across. This is likely to be as true for coercion-reduction innovations as any other new healthcare practice with a convincing evidence base. In short it could be said that we know the answer to the question ‘what works?’ but we do not yet know the answer to the next question, ‘how do we get people to use what works every day?’

Implementation science (IS) is increasingly recognised as a crucial source of knowledge for bridging this gap between research and practice. There are likely to be certain core factors which influence implementation in many healthcare fields, such as motivation amongst individual staff and the culture of the whole organisation [119], and there may be additional factors which are specific to the implementation of coercion-minimisation interventions. At this time, a range of implementation strategies have been articulated, which can be considered as part of a practical but robust model for maximising the adoption of a coercion-reduction programme in practice. These potential strategies can be summarised under a number of thematic clusters, including the provision of interactive assistance, tailoring the intervention to the context and developing stakeholder inter-relationships [120].

Adopting novel ways of working requires substantial effort by healthcare staff on top of the intensive workload that they already carry in front-line services. Learning and successfully implementing all the components of a complex intervention, such as Six Core Strategies or Safewards, requires significant and ongoing support from management in any organisation and genuine ‘buy-in’ by those tasked with changing their practices. The same can be said of implementing the components of the integrated community-based network of services that is necessary to reduce involuntary admissions, a process that requires complex structural reforms and profound changes of practices. Adoption of less complex measures such as shared decision-making needs fewer resources but requires a challenging shift amongst staff in terms of power and responsibility for the consequences of risky behaviour in highly volatile crisis situations. Currently, the models and frameworks available from IS based on investigations of many different types of organisational change in healthcare and beyond are rarely used when attempting to embed innovations into everyday practice [121]. So, the next step is learning from IS and using its knowledge to underpin service transformation in both high-income countries and those with fewer resources. If the emerging innovations discussed above remain both effective and genuinely acceptable to both clinicians and patients in mental health services, we can be confident there is a road map for reducing coercion to its absolute minimum. Beyond that, the total abolition of coercion via zero tolerance will require the wider public in democratic countries around the world to transform their own attitudes to mental disorders and provide the necessary resources to enable clinicians to do their jobs free of the need for compulsion.

## 5. Conclusions

In the last few decades, there have been important conceptual developments in the way we approach the nature and causes of mental illness, the factors involved in disabilities and the organization of services. Important paradigm shifts took place and are still occurring, moving from a custodial paradigm to a care paradigm, then to a recovery paradigm, and now to a human rights paradigm. The goal of reducing coercion continues to face important obstacles. However, because of the increasing awareness of the risks involved in using coercive practices, the increasing knowledge of the current availability of alternatives to coercion, and the challenges associated with implementation, never have there been such favourable and fertile conditions to reach these goals. The project of a more humane and multi-faceted conception of mental health patients and their care continues to advance and is going through a particularly exciting phase.

## Figures and Tables

**Table 1:** Some promising interventions for the reduction of coercion in mental health services.

Community Services	Inpatient Services	Both Service Levels
Advance directives/advance planning ^2^	**Six Core Strategies** ^1,3^	Medication discontinuance ^3^
**Joint crisis plans** ^2^	**Safewards** ^1,3,^**	Peer support ^3^
Crisis plan/card ^2^	Talk First ^1^	Recovery models ^3^
Early warning symptoms ^2^	ResTrain Yourself ^1^	Representation agreements^3^
Community Treatment Order ^2^	Scottish Patient Safety Programme ^1^	**Shared decision-making** ^2,3,^**
Treatment adherence therapy ^2^	Seclusion Reduction Programme ^1^	Trauma-informed ^3^
Financial incentives ^2^	Sensory Modulation ^1^	
Crisis Resolution Team ^2^	**Restraint education** ^2^	
Integrated treatment ^2^		
Club House ^3^		
Hearing Voices Network ^3^		
Respite houses ^3^		
Soteria House ^3,^**		
Trialogue ^3^		

^1^ Cited as an ‘intervention family’ in four or more empirical studies analysed by Baker et al. [101]—any evaluation methodology; ^2^ Cited as an intervention tested in at least one RCT by Barbui et al. [87]; ^3^ Cited as a ‘prominent measure or approach’ which has been implemented ‘internationally’ or in at least five countries by Gooding et al. [104]; ** Intervention implemented at least once in a low- or middle-income country; **Bold**: intervention with evidence of effectiveness in at least one RCT.

## Data Availability

Not applicable.
